# Supplementary material for: Association between oral microbiome and seven types of cancers in East Asian population: a two-sample Mendelian randomization analysis
Source: Front Mol Biosci. 2023 Nov 21;10:1327893. doi: 10.3389/fmolb.2023.1327893 (PMC10702768; doi:10.3389/fmolb.2023.1327893)
Supplement: Supplementary file 1 [file DataSheet1.ZIP › Supplementary Materials/MR plots of saliva/saliva═╝/Prostate cancer/pheno.3154_to_prostate cancer_leave_one_out.pdf]

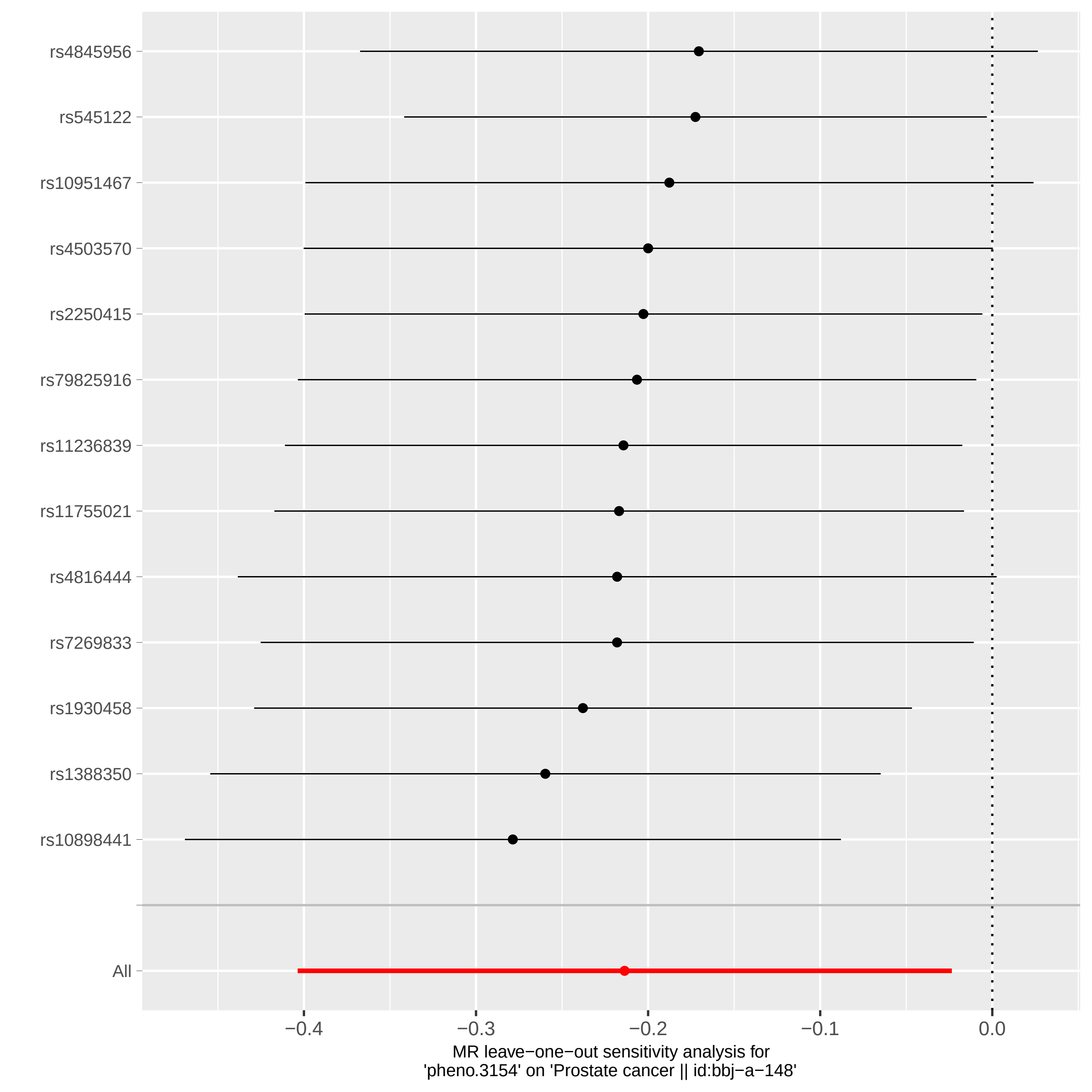
rs4845956
rs545122
rs10951467
rs4503570
rs2250415
rs79825916
rs11236839
rs11755021
rs4816444
rs7269833
rs1930458
rs1388350
rs10898441
All
−0.4
−0.3
−0.2
−0.1
0.0
MR leave−one−out sensitivity analysis for
'pheno.3154' on 'Prostate cancer || id:bbj−a−148'
